# Omalizumab in symptomatic therapy of laryngeal oedema and urticaria attacts in a patient with post operative pulmonary carsinoid tumor

**DOI:** 10.1186/1939-4551-8-S1-A246

**Published:** 2015-04-08

**Authors:** Arzu Didem Yalcin, Kaya Suer

**Affiliations:** 1 Near East University, Cyprus

## 

Omalizumab has been investigated in various other conditions including chronic urticaria (CU), perennial and seasonal allergic rhinitis (AR), pruritic bullous pemphigoid, latex allergy, peanut allergy, idiopathic anaphylaxis, hyper-IgE syndrome, chronic rhinosinusitis, interstitial cystitis, aspirin sensitivity, mastocytosis, eosinophilic gastroenteritis and atopic dermatitis. Most patients with chronic urticaria have an autoimmune cause: some patients produce IgE autoantibodies against autoantigens, such as thyroperoxidase or doublestranded DNA, whereas other patients make IgG autoantibodies against FcεRI, IgE, or both, which might chronically activate mast cells and basophils.I had a male patients with food allergy with pulmonary carsinoid tumor, aged 23. Autologous Serum Skin Test, Anti Nuclear Antibody,and hepatitis markers (HBsAg, HBsAb, anti HCV HIV, thyroid antibodies were negative in patients. Liver, thyroid, and renal function tests, serum IgG, IgA, IgM, levels were within normal ranges. Skin prick tests (SPTs) were highly positive for kiwi, tometo, fish, orange The specific IgE levels were correlated with the SPTs. Total IgE level were 960 IU/L (normal range:0-100 IU/L).A mass was defined on lober lobe of left lung on computerized tomography. PET CT SUVmax:6. The patient was operated. In postoperative period, he had recurrent laryngeal oedema and urticaria attacks. Omalizumab treatment planned because of the patient was resistant to antihistaminics and steroids.

For the very first time, we used omalizumab in symptomatic therapy of recurrent laryngeal oedema and urticaria attacts in a patient with post operative pulmonary carsinoid tumor for eight months. During the four years of follow-up, no reccurrens was noted in carsinoid tumor. Control PET CT and CT results revealed normal findings.After omalizumab treatment, symptoms were decreased. Oral antihistaminics and mast cell stabilizing drugs used for treatment afterwards. Oral steroid was given only once.

Resent sutudies show that, Pooled data analysis revealed that a causal relationship between omalizumab therapy and malignancy is unlikely.

